# Predicting steady‐state endoxifen plasma concentrations in breast cancer patients by *CYP2D6* genotyping or phenotyping. Which approach is more reliable?

**DOI:** 10.1002/prp2.646

**Published:** 2020-08-19

**Authors:** Milena Gusella, Felice Pasini, Barbara Corso, Laura Bertolaso, Giovanni De Rosa, Cristina Falci, Yasmina Modena, Carmen Barile, Donatella Da Corte Z, AnnaPaola Fraccon, Silvia Toso, Elisabetta Cretella, Antonella Brunello, Caterina Modonesi, Romana Segati, Cristina Oliani, Nadia Minicuci, Roberto Padrini, for the Italian TAM Group

**Affiliations:** ^1^ Oncology Unit AULSS5 Polesana Rovigo Italy; ^2^ Oncology Unit Casa di Cura Pederzoli Peschiera del Garda Italy; ^3^ National Research Council Neuroscience Institute Padova Italy; ^4^ Clinical Pharmacology Unit of the Department of Medicine (DIMED) University of Padova Padova Italy; ^5^ Oncology Unit 2 Istituto Oncologico Veneto (IOV) IRCCS Padova Padova Italy; ^6^ Oncology Unit Ospedale di Belluno Belluno Italy; ^7^ Oncology Unit Ospedale di Adria Polesana Italy; ^8^ Oncology Unit Ospedale di Bolzano Bolzano Italy; ^9^ Oncology Unit 1 Istituto Oncologico Veneto (IOV) IRCCS Padova Padova Italy; ^10^ Oncology Unit Ospedali Riuniti Padova Sud Padova Italy; ^11^ Oncology Unit Ospedale di Feltre Feltre Italy

**Keywords:** breast cancer, *CYP2D6*, dextromethorphan, endoxifen

## Abstract

In previous studies, steady‐state Z‐endoxifen plasma concentrations (ENDOss) correlated with relapse‐free survival in women on tamoxifen (TAM) treatment for breast cancer. ENDOss also correlated significantly with *CYP2D6* genotype (activity score) and *CYP2D6* phenotype (dextromethorphan test). Our aim was to ascertain which method for assessing *CYP2D6* activity is more reliable in predicting ENDOss. The study concerned 203 Caucasian women on tamoxifen‐adjuvant therapy (20 mg q.d.). Before starting treatment, *CYP2D6* was genotyped (and activity scores computed), and the urinary log(dextromethorphan/dextrorphan) ratio [log(DM/DX)] was calculated after 15 mg of oral dextromethorphan. Plasma concentrations of TAM, N‐desmethyl‐tamoxifen (ND‐TAM), Z‐4OH‐tamoxifen (4OH‐TAM) and ENDO were assayed 1, 4, and 8 months after first administering TAM. Multivariable regression analysis was used to identify the clinical and laboratory variables predicting log‐transformed ENDOss (log‐ENDOss). Genotype‐derived *CYP2D6* phenotypes (PM, IM, NM, EM) and log(DM/DX) correlated independently with log‐ENDOss. Genotype‐phenotype concordance was almost complete only for poor metabolizers, whereas it emerged that 34% of intermediate, normal, and ultrarapid metabolizers were classified differently based on log(DM/DX). Multivariable regression analysis selected log(DM/DX) as the best predictor, with patients’ age, weak inhibitor use, and *CYP2D6* phenotype decreasingly important: log‐ENDOss = 0.162 ‐ log(DM/DX) × 0.170 + age × 0.0063 ‐ weak inhibitor use × 0.250 + IM × 0.105 + (NM + UM) × 0.210; (*R* ^2^ = 0.51). In conclusion, log(DM/DX) seems superior to genotype‐derived *CYP2D6* phenotype in predicting ENDOss.

**What is already known about this subject**

- Endoxifen is the active metabolite of tamoxifen, which is responsible for most of its anti‐estrogen activity.
- Steady‐state endoxifen concentrations (ENDOss) >5.97 ng ml‐1 correlate with relapse‐free survival of breast cancer patients.
- *CYP2D6* phenotype inferred from *CYP2D6* genotype and dextromethorphan/dextrorphan metabolic ratio [log (DM/DX)] correlate with ENDOss.

**What this study adds**

- An algorithm including log(DM/DX), patient's age and weak inhibitor use predicts 48% of log‐ENDOss variability.
- *CYP2D6* phenotypes have a weaker predictive power than log(DM/DX) (*R* ^2^ = 0.41).
- The model based on log(DM/DX) may identify patients who will have ENDOss <5.97 ng ml‐1, and consequently require a higher starting dose of tamoxifen.

## 1 INTRODUCTION

Tamoxifen (TAM) is a selective estrogen receptor antagonist used as adjuvant therapy to prevent estrogen‐receptor‐positive breast cancer recurrence. TAM is de facto a prodrug because its anti‐estrogen activity is 30‐100 times less than that of its metabolites Z‐4OH‐tamoxifen (4OH‐TAM) and Z‐endoxifen (ENDO).^1^, ^2^ ENDO is considered the most effective metabolite in vivo, with plasma concentrations 5‐10 times higher than 4OH‐TAM.^3^ Two well‐powered trials found ENDO plasma concentrations correlated with recurrence risk.^4^, ^5^ Madlensky et al ^4^ reported that women with ENDO concentrations >5.97 ng ml‐1 (>16 nM) had a 30% lower relative risk of breast cancer recurrence. Saladores et al ^5^ found ENDO levels <5.2ng ml‐1 (<14 nM) associated with a shorter relapse‐free survival (RFS) compared with >13.0 ng ml‐1 (>35 nM). Hence the suggestion that monitoring ENDO concentrations can be used to individualize adjuvant TAM therapy.^6^, ^7^

**Figure 1:**
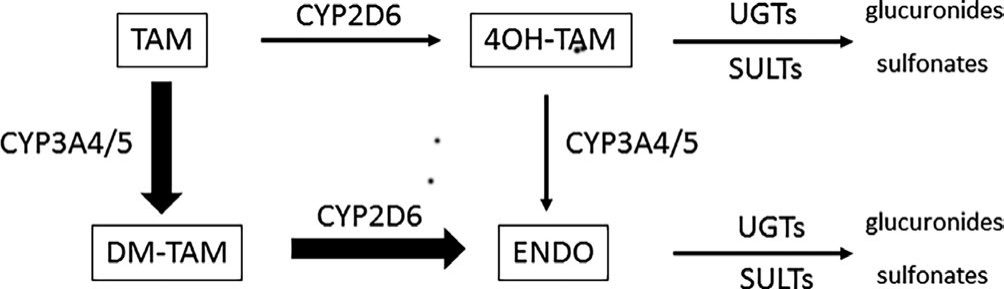
Main metabolic pathways of tamoxifen. TAM: tamoxifen; DM‐TAM: desmethyl‐tamoxifen; 4OH‐TAM: Z‐4OH‐tamoxifen; ENDO: Z‐endoxifen; UGTs: UDP‐glucuronosyl transferases; SULTs: sulfotransferases

An alternative strategy involves measuring predictors of steady‐state ENDO levels (ENDOss) before starting TAM therapy. While several enzymes contribute to ENDO formation (CYP3A4/5, CYP2C9, CYP2C19, CYP1A2) and elimination (UGTs, SULTs), the main metabolic pathway is ENDO formation from N‐desmethyl‐tamoxifen (ND‐TAM) by the cytochrome *CYP2D6* ^8^ (Figure 1). *CYP2D6* activity has been estimated indirectly by combining the several *CYP2D6* allelic variants with a different gene expression,^9^ or calculated directly from the dextromethorphan (DM)/dextrorphan (DX) urinary metabolic ratio [log(DM/DX)].^10^, ^11^ Both methods can predict ENDOss. *CYP2D6* phenotyping is considered superior to genotyping because non‐genetic factors like age, drug‐drug interactions, or co‐morbidities can affect phenotype (phenoconversion phenomenon),^12^ but the two methods’ performance had yet to be compared directly.

Our primary aim was to ascertain which method ‐ *CYP2D6* genotyping or phenotyping ‐ can predict ENDOss more accurately. The findings presented here are part of an ongoing prospective trial (TAM study) to correlate ENDOss with breast cancer recurrence.

## 2 METHODS

### 2.1 Patients and study design

This study concerned 203 Caucasian women with estrogen‐receptor‐positive breast cancer (stage IA 67.2%, IIA 23.2%, IIB 6.8%, and IIIA 2.8%) on TAM adjuvant therapy (20 mg q.d.) involved in a trial enrolling patients from 20 oncology units in Northern Italy.

Before starting TAM, blood samples were drawn for *CYP2D6* genotyping. *CYP2D6* phenotyping was done as follows: 15 mg of oral dextromethorphan were administered at 10 PM, then urine was collected overnight until 8 AM, when a sample was frozen at −20°C until analysis of DM and DX concentrations (see below).

One, 4, and 8 months after starting TAM, blood was sampled before a drug dose to assay plasma concentrations of TAM, ND‐TAM, 4OH‐TAM, and ENDO. All other routine procedures were completed according to local clinical practice.

The study protocol was approved by the Ethics Committee of Rovigo Hospital (Italy) and all participants gave their written informed consent.

### 2.2 Plasma assay of ENDO, 4OH‐TAM, ND‐TAM, and TAM

Tamoxifen and its metabolites were analyzed in patients’ plasma using a validated high‐performance liquid chromatography (HPLC) method,^13^ with partial adaptations. Briefly, blood was centrifuged within an hour of sampling and plasma was stored at −20°C until analysis. One mL of plasma was alkalinized with 1 ml‐glycine/NaOH buffer (1M, pH: 11.3) and extracted with 7 ml of hexane/2‐propanol (95:5, v:v). After centrifugation, the supernatant was collected, dried under nitrogen stream and re‐suspended in 200 µl of mobile phase, then 30 µl were injected in a HPLC system (mod. 1515; Waters Corp, Milford, MA) for separation in a C18 column (Kromasil 100‐3.5C18, 150x4.6 mm). All compounds were then converted to more fluorescent derivatives with an UV photochemical reactor (PHRED, Aura Industries, NY, USA) using a 254 nm wavelength, then detected with a fluorescence detector (mod. 2487; Waters Corp, Milford, MA) with excitation and emission wavelengths set at 256 and 380 nm, respectively. The mobile phase consisted of 40% acetonitrile in phosphate buffer (20 mM, pH 3.0), with a flow rate of 1ml min‐1. Calibration curves were obtained with plasma from healthy volunteers by adding known concentrations of ENDO (range 1.25‐20 ng ml‐1), 4OH‐TAM (0.625‐10 ng ml‐1), ND‐TAM (25‐400 ng ml‐1), and TAM (25‐ 400 ng ml‐1). Two internal standards were used: propranolol for TAM; and ND‐TAM (0.5 µg ml‐1) and verapamil for ENDO and 4OH‐TAM (0.25 µg ml‐1). Calibration curves were considered acceptable if *R* ^2^ ≥ 0.99. Precision, accuracy, and quantification limits are shown in Appendix 1.

### 2.3 *CYP2D6* genotyping procedure

Germline DNA was isolated from blood using Wizard Genomic DNA Purification Kit (Promega) according to the manufacturer's recommendations.

Samples were analyzed for six polymorphisms and a full gene deletion, accounting for most of the clinically significant variants of *CYP2D6* in Caucasian populations.^14^

Genotyping was conducted using PCR/RLFP‐based methods for CYP2D6*3 (2549 A del, rs35742686), CYP2D6*4 (1846G>A, rs3892097), CYP2D6*6 (1707T del, rs5030655), CYP2D6*9 (2615_2617del AAG, rs5030656), CYP2D6*10 (100C>T, rs1065852), with digestion by MspI, BstN1, BtsI, MboII, and HphI, respectively, as in other studies.^15^, ^16^, ^17^, ^18^

Allele *41 (G2988A, rs28371725) was detected using denaturing HPLC. Specific primers were designed with Primer3 software ^19^ and confirmed with Human Genome Browser in silico tools as follows: fw 5’‐GAGCCCATCTGGGAAACAGT‐3’ and rv 5’‐CCTCCTATGTTGGAGGAGGTC‐3’. PCR was performed with 1U of Hot Start DNA polymerase AmpliTaq Gold (Applied Biosystems) in a final volume of 50 µL; the annealing temperature was 58°C, for 38 cycles. The optimal melting temperatures for SNP detection was experimentally determined as 62.8°C. Each sample was run alone and with a plasmid positive control (containing the *CYP2D6* 2988A variant, obtained with the QuikChange Site‐Directed Mutagenesis kit by Agilent Technologies) for 8 min with a gradient mobile phase consisting of Buffers A (triethyl ammonium acetate) and B (triethyl ammonium acetate and acetonitrile) at a flow rate of 0.9 ml min‐1. Retention times of 4.5 and 5 min were associated with heteroduplex (2988G/A) and homoduplex (2988A) profiles, detecting wild‐type and mutant alleles, respectively. Variant genotypes were verified by direct Sanger sequencing (CEQ2000XL, Beckman Coulter). Full *CYP2D6* deletion (CYP2D6*5) analysis was conducted with a long‐range PCR using the DyNAzyme II DNA Polymerase kit (Thermo Fisher Scientific) according to the manufacturer's instructions and a 1% agarose gel run, as described by Sistonen et al.^20^

The *CYP2D6* activity score was calculated according to the Clinical Pharmacogenetic Implementation Consortium and Dutch Pharmacogenetics Working Group criteria,^21^ which assigned scores of 0, 0.25, 0.5, 1, or 2 to each allele based on their relative activity compared with the wild type (=1), as follows: *3, *4, *5, *6 = 0; *10 = 0.25; *9, *41 = 0.5; no variant alleles = 1; and *1 × 2N = 2. The sum of the activity scores for each allele (AS) was translated into the following *CYP2D6* phenotypes: ultrarapid metabolizers (UM), AS > 2.25; normal metabolizers (NM), 1.25 ≤ AS ≤2.25; intermediate metabolizers (IM), 0 < AS <1.25; poor metabolizers (PM), AS = 0.

### 2.4 Urinary DM and DX assay

Urinary DM and DX were tested using HPLC according to Flores‐Péres et al,^22^ with slight modifications. Before the extraction procedure, 0.5 ml of urine was hydrolyzed overnight at 37°C by adding 0.5 ml of a solution of β‐glucuronidase (2000 U ml‐1) in acetate buffer (pH 5). This step was necessary because most DX in urine is in the form of glucuronide. Then 500 mL of hydrolysate were spiked with 25 µl of a 0.1 mg ml‐1 levallophan solution (as internal standard) and 500 µL of carbonate buffer (pH 9.2) were added. Extraction was done with 3.5 mL of a hexane‐butanol mixture (95:5, v/v) in a shaker rotated for 10 minutes. After centrifugation at 855 g for 5 minutes, the organic phase was separated and evaporated to dryness at 55°C under gentle nitrogen stream. The residue was reconstituted with 1 ml of mobile phase and 10 µl were injected in the HPLC column (Kromasil® 100‐5 phenyl, 250 × 4.6mm), thermostated at 30°C. The mobile phase, a mixture of acetonitrile and acetic acid 1% + triethylamine 0.1% (35:65), was fluxed at 1 ml min‐1 with an isocratic pump (mod. 1515; Waters Corp, Milford, MA). The effluent was analyzed with a fluorescence detector (mod. 2487; Waters Corp, Milford, MA) set at excitation and emission wavelengths of 275 nm 310 nm, connected with Empower 3 software (Waters Corp Milford, MA).

Calibration curves were prepared by adding increasing volumes of the working solutions of dextromethorphan hydrobromide (0.1 mg ml‐1 = 270mM) and dextrorphan tartrate (0.1 mg ml‐1 = 245mM) to distilled water to obtain concentrations in the range of 0.25‐10 μg ml‐1. Within this range, the curves were linear with a coefficient of determination (*R* ^2^) always > 0.99. Precision, accuracy, and quantification limits are shown in Appendix 1.

### 2.5 *CYP2D6* phenotyping procedure

The logarithm of the ratio of urinary DM to DX molar concentrations [log(DM/DX)] was taken as a measure of *CYP2D6* activity. Patients were classified as poor metabolizers (PM), intermediate metabolizers (IM), extensive metabolizers (NM), or ultra‐rapid metabolizers (UM) according to their log(DM/DX) ratio, as follows: PM ≥ −0.52; IM <−0.52 and ≥ −1.52; NM, <−1.52 and ≥‐2.52; UM <−2.52.^10^

Since ND‐TAM is metabolized to ENDO by the cytochrome *CYP2D6*, the logarithm of the ratio of ND‐TAM to ENDO plasma concentrations [log(ND‐TAM/ENDO)] was considered another independent measure of *CYP2D6* activity.

### 2.6 Statistical analysis

In the tables, continuous variables are presented as means ± standard deviations (unless otherwise stated), and categorical variables as absolute numbers and percentages.

Continuous variables with a normal distribution were compared with Student's *t* test. One‐way ANOVA was used for comparing more than two independent groups, followed by Bonferroni post‐hoc tests for pairwise comparisons, and the test for linear trend, as needed. Two‐way ANOVA was used to compare repeated measures from the same patient. The homoscedasticity assumption was verified with the Bartlett and Levene test. Equivalent non‐parametric tests (the Mann‐Whitney U, Kruskal‐Wallis and Friedman tests) were used whenever applicability conditions were not met. Categorical data frequencies were examined using Pearson's chi‐square and Fisher's exact test, as appropriate.

ENDOss was calculated as the mean of ENDO concentrations at 4 and 8 months, if both measurements were available, or at 4 months otherwise. A log‐transformation was applied (log‐ENDOss) to achieve a normal distribution of ENDOss.

The following independent variables were used in the regression analyses: age (years), body weight (kg), body surface area (BSA; calculated with the Haycock formulae, m^2^), body mass index (BMI; kg m^2^‐1), log(DM/DX), concomitant use of *CYP2D6* weak inhibitors, and *CYP2D6* activity score. *CYP2D6* activity scores were translated into four phenotypes (UM, NM, IM, PM), according to the updated CPIC guidelines.^21^ Since only one patient was classified as UM (genotype 1 × 2N*1), she was included in the NM group.

First, univariable linear regression analyses were run, taking one independent variable at a time. Then, a stepwise multivariable forward regression was conducted (*P* < .05 for variable inclusion, and *P* < .15 for variable removal) to select the best log‐ENDOss prediction model. Multicollinearity was checked using the tolerance and the variance inflation factor (VIF); variables with a tolerance <0.4 (VIF > 2.5) were discarded from the analysis.

Possible confounding effects were investigated with all variables excluded by the stepwise selection. Residuals analysis was performed to examine the models’ goodness of fit and adherence to the regression assumptions. The validity of the final model was assessed by measuring the *R* ^2^ coefficient and the mean absolute and percentage errors (MAE and MAPE) of the ENDOss predicted.

Pearson's correlation coefficients (r) were used to test the relationship between log(DM/DX) and log‐ENDOss, and between log(DM/DX) and log(NDT/ENDO).

A receiver operating characteristics (ROC) analysis was used to identify the threshold for the log(DM/DX) ratio associated with ENDOss <5.97 ng ml‐1, and the area under the ROC curve (AUC) was estimated.

All statistical analyses were performed using STATA SE, version 12.1 (Stata Statistical Software, College Station, TX: StataCorp LP), setting the level of significance at 0.05.

## 3 RESULTS

### 3.1 Patients’ characteristics

Of the population of 203 women, only 164 were suitable for multivariable regression analyses as all the independent variables were available. Table 1 summarizes patients’ characteristics for the whole group and for the regression group. No patients were taking strong *CYP2D6* inhibitors, while 12 in the whole group and 9 in the regression group were taking weak inhibitors (citalopram, sertraline, duloxetine, venlafaxine). There were no statistically significant differences between the variables considered in the two groups, except for age (*P* = .0083), and menopausal status (*P* = .023).

**Table 1:** Patients’ characteristics in the whole sample and in the group for regression analysis

Variable	Whole sample (N = 203)	Sample for regression analysis (N = 164)
Age (years), mean ± SD [range]	56.2 ± 11.7 [29‐89]	57.2 ± 11.2 [33‐89]
Body weight (kg), mean ± SD [range]	67.4 ± 13.5 [42 ‐ 115]	67.8 ± 13.9 [43‐115]
Body surface area (m^2^), mean ± SD [range]	1.75 ± 0.20 [1.33‐2.44]	1.75 ± 0.20 [1.36‐2.44]
BMI (kg m^2^‐1), mean ± SD [range]	25.7 ± 5.0 [15.8‐42.9]	25.9 ± 5.1 [15.8‐42.9]
In menopause, n (%)		
Yes	147 (73%)	126 (77%)
No	53 (27%)	38 (23%)
Weak inhibitor use, n (%)		
Yes	12 (6%)	9 (5%)
No	191 (94%)	155 (95%)
ENDO concentration (ng ml‐1) after 1 month, mean ± SD [range]	8.05 ± 4.85 [1.20‐27.00]	8.13 ± 5.05 [1.20‐27.00]
ENDO concentration (ng ml‐1) in steady state, mean ± SD [range]	10.57 ± 6.83 [1.60‐40.41]	10.69 ± 6.88 [1.60‐40.41]
Log(DM/DX), mean ± SD [range]	−1.59 ± 0.89 [−3.08‐1.39]	−1.61 ± 0.87 [−3.08‐1.39]
*CYP2D6* phenotype, n (%)		
PM	15 (8.1%)	14 (8.6%)
IM	64 (34.2%)	53 (32.3%)
NM	107 (57.2%)	96 (58.5%)
UM	1 (0.5%)	1 (0.6%)

Abbreviations: BMI, Body Mass Index; DM, dextromethorphan; DX, dextrorphan; ENDO, Z‐endoxifen plasma concentrations; IM, intermediate metabolizer; NM, normal metabolizer; PM, poor metabolizer; UM, ultrarapid metabolizer.

### 3.2 ENDO, 4OH‐TAM, ND‐TAM and TAM plasma concentrations

**Figure 2:**
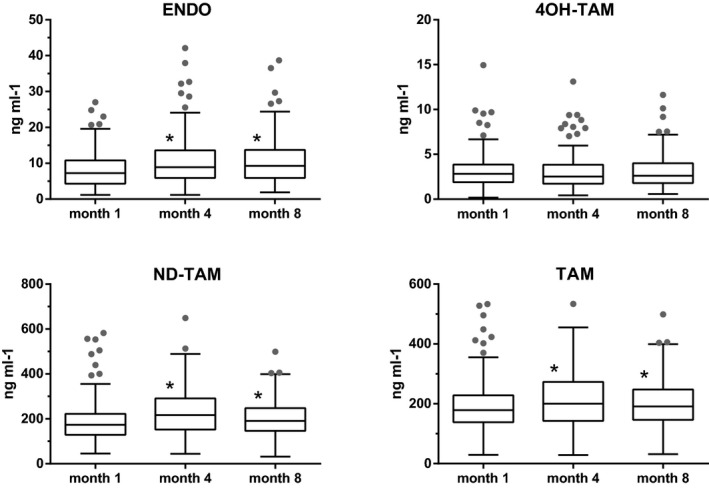
Box and whisker plots (circles are outliers) of plasma concentrations of endoxifen (ENDO), 4OH‐tamoxifen (4OH‐TAM), N‐desmethyl‐tamoxifen (ND‐TAM), and tamoxifen (TAM) after 1, 4, and 8 months. Asterisks indicate significant differences from values at 1 month

Figure 2 shows the median plasma concentrations (box and whisker plots) of all compounds during the follow‐up. All measures showed a wide inter‐subject variability.

Four separate two‐way repeated‐measures ANOVAs were run to identify any differences in the concentrations of the four compounds at the different times (1, 4 and 8 months). The results showed that mean ENDO, ND‐TAM and TAM concentrations rose significantly from the first to the fourth month, then remained stable (for all three compounds, comparisons were significant [*P* < .0001] for month 1 vs month 4, and for month 1 vs month 8), while 4OH‐TAM concentrations remained stable throughout the observation period. The mean absolute difference in ENDO concentrations between month 4 and month 8 was 2.5 ± 2.6 ng mL‐1 (mean change: +8%, n.s.).

### 3.3 Log(DM/DX) and *CYP2D6* phenotype

**Figure 3:**
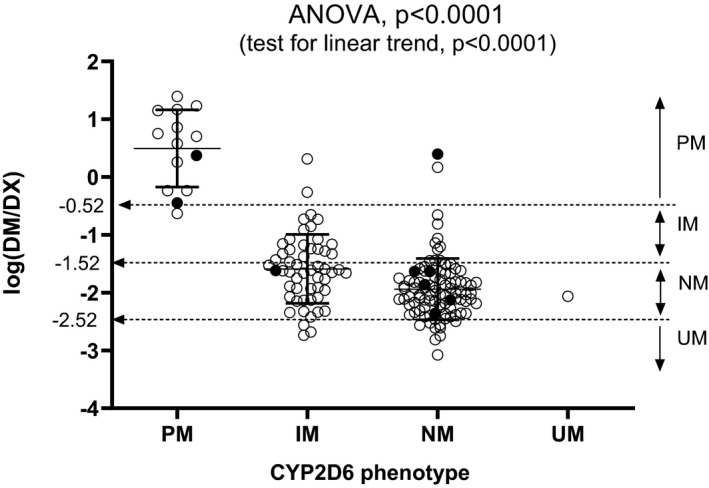
Distribution of log(DM/DX) across the four *CYP2D6* phenotypes. Filled symbols refer to the concentrations in users of weak inhibitors. Horizontal lines represent means and vertical bars 95% confidence intervals. Dashed arrows indicate the log(DM/DX) cut‐offs that separate poor (PM), intermediate (IM), extensive (EM), and ultra‐rapid metabolizers (UM)

One‐way ANOVA followed by testing for linear trends showed a significant difference in the mean log(DM/DX) values across groups identified by *CYP2D6* phenotype (*P* < .0001) (Figure 3). These log(DM/DX) values varied considerably within each group, however, indicating that CYP2D6 activity inferred from *CYP2D6* genotype cannot accurately predict the phenotype. In fact, 34% of patients classified according to the *CYP2D6* genotype ^21^ did not match the phenotype assessed with the log (DM/DX) classification system.^10^

### 3.4 ENDOss, Log(DM/DX), and *CYP2D6* phenotype

**Figure 4:**
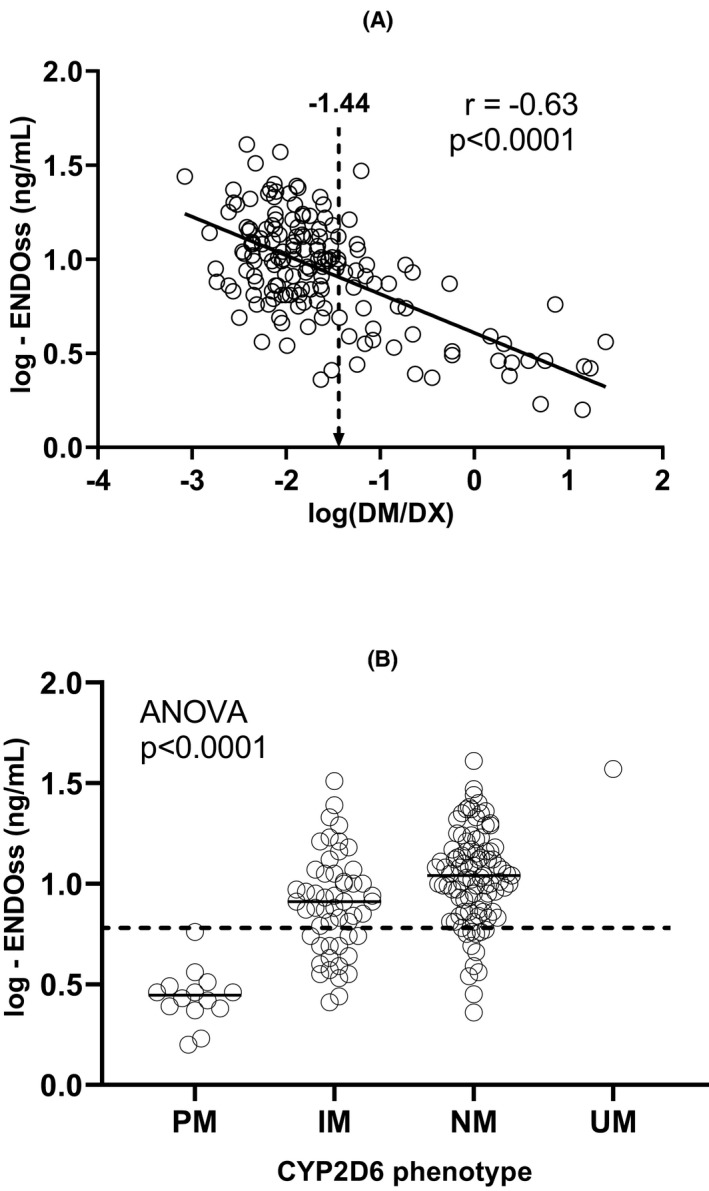
Panel (a): correlation between log(DM/DX) and log‐transformed steady‐state endoxifen concentrations (log‐ENDOss). The dashed arrow indicates the best log(DM/DX) cut‐off associated with ENDOss < 5.97 ng ml‐1 Panel (b): distribution of log‐ENDOss across the four *CYP2D6* phenotypes. The dashed line indicates the log‐ENDOss cut‐off of 0.779, corresponding to ENDOss of 5.97 ng ml‐1.

Log‐ENDOss correlated inversely with log(DM/DX) (r = 0.63; *P* < .0001) (Figure 4, panel a) and one‐way ANOVA showed a rising trend of log‐ENDOss in parallel with *CYP2D6* phenotype (significant comparisons: PM vs IM, NM + UM; and IM vs NM + UM; *P* < .0001) (Figure 4, panel b). Similar correlations emerged for steady‐state 4OH‐TAM concentrations (4OH‐TAMss), whereas steady‐state DM‐TAM levels (DM‐TAMss) correlated directly with log(DM/DX), and inversely with *CYP2D6* phenotype (data not shown). TAM concentrations did not correlate with *CYP2D6* activity markers.

**Figure 5:**
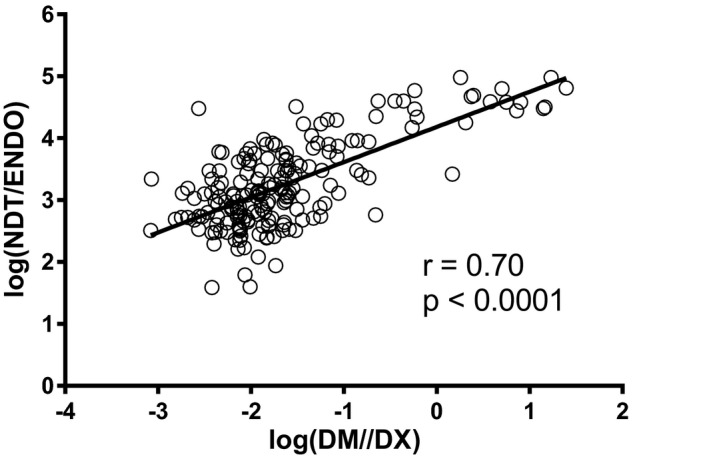
Correlation between urinary log(DM/DX) ratio and plasma log(ND‐TAM/ENDO) ratio measured after 1 month of therapy

Of note, urinary log(DM/DX) correlated significantly with plasma log(ND‐TAM/ENDO) at 1 month (*r* = 0.70; *P* < .0001), indicating that both ratios reflect *CYP2D6* metabolic activity (Figure 5).

Considering the ENDOss concentration of 5.97 ng ml‐1 indicated by Madlensky et al ^4^ as the threshold for a favorable clinical outcome, our data show that all patients with an activity score of 0 had sub‐therapeutic ENDO levels (Figure 4, panel b). ROC analysis identified a cut‐off for log(DM/DX) of −1.445 beyond which most patients had ENDOss ≤5.97 ng ml‐1 (0.776 in log‐scale), with 89% sensitivity and 67% specificity (AUC = 0.82, 95% confidence interval: 0.74‐0.91, *P* < .001) (Figure 4, panel a).

### 3.5 Univariable e multivariable analyses

On univariable linear regression, the following variables significantly predicted log‐ENDOss: log(DM/DX) (*R* ^2^ = 0.39); *CYP2D6* phenotype (*R* ^2^ = 0.37); weak inhibitor use (*R* ^2^ = 0.055); and body surface area (*R* ^2^ = 0.032) (Table 2). After multicollinearity checking, body surface area was discarded from subsequent analyses due to its collinearity with BMI.

**Table 2:** Intercepts, β coefficients and significance levels obtained by univariable regression analyses

Variables	Intercept (95% CI)	β Coefficients (95% CI)	*P*‐value	*R* ^2^
Log(DM/DX)	0.61 (0.54 to 0.68)	−0.21 (−0.25 to −0.17)	<.0001	39.25
*CYP2D6* phenotype	0.44 (0.32 to 0.56)			
IM		0.46 (0.32 to 0.60)	<.0001	34.73
NM + UM		0.60 (0.47 to 0.73)	<.0001	
Weak inhibitor use	0.96 (0.91 to 1.00)	−0.29 (−0.48 to −0.11)	.002	5.53
Body surface area (m^2^)	1.39 (1.00 to 1.77)	−0.25 (−0.47 to −0.037)	.022	3.19
BMI (kg m^2^‐1)	1.05 (0.83 to 1.28)	−0.0043 (−0.013 to 0.004)	ns (.33)	0.59
Age, (years)	0.89 (0.66 to 1.12)	0.0009 (−0.003 to 0.005)	ns (.65)	0.12

The stepwise multivariable regression analysis identified log(DM/DX), patient's age, weak inhibitor use, and *CYP2D6* phenotype as significant independent predictors of log‐ENDOss, ruling out BMI:

(1) $$\begin{array}{cc} \log‐\text{ENDOss} & =0.162‐\log\left(\text{DM}/\text{DX}\right)\times 0.170+\text{age}\\ & \;\times 0.0063‐\text{weak inhibitor use}\times 0.250\\ & \;+\text{IM}\times 0.105+\left(\text{NM}+\text{UM}\right)\times 0.210 \end{array}$$

*R* ^2^ = 0.510; MAE = 0.16 ng ml‐1; MAPE = 19.9%

Log(DM/DX) and *CYP2D6* phenotype were collinear so they were alternately removed from the regression to see which model performed better:

(2) $$\begin{array}{cc} \log‐\text{ENDOss} & =0.225‐\log\left(\text{DM}/\text{DX}\right)\times 0.223\\ & \;+\text{age}\times 0.0065‐\text{weak inhibitor use}\times 0.235 \end{array}$$

*R* ^2^ = 0.478; MAE = 0.16 ng ml‐1; MAPE = 21.2%

(3) $$\begin{array}{cc} \log‐\text{ENDOss} & =0.218+\text{IM}\times 0.444+\left(\text{NM}+\text{UM}\right)\times 0.608\\ & \;+\text{age}\times 0.0041‐\text{weak inhibitor use}\times 0.265 \end{array}$$

*R* ^2^ = 0.410; MAE = 0.17 ng ml‐1; MAPE = 21.8%

Equation 2, which included log(DM/DX), yielded a higher *R* ^2^ than Equation 3, with small changes in MAE and MAPE.

To translate these models into clinically relevant information, linear ENDOss were predicted for each patient by transforming the results of Equations (1), (2), (3) into the corresponding anti‐logarithms. Tables 3, 4, 5 show the partial variances (*R* ^2^) explained by each variable in Equation 1, 2, and 3. Table 6 shows the MAEs and MAPE (± SD, range) of the ENDOss obtained with each equation.

**Table 3:** β Coefficients and significance levels of variables significantly associated with log‐transformed ENDOss, by multivariable regression analysis (Equation 1)

Variable	β Coefficient (95% CI)	*P*‐value	Partial *R* ^2^
Intercept	0.162 (−0.047 to 0.371)	.127	—
Log(DM/DX)	−0.170 (−0.228 to −0.111)	<.0001	39.25
Age (years)	0.0063 (0.003 to 0.009)	<.0001	5.13
Weak inhibitor use	−0.250 (−0.389 to −0.110)	.001	3.46
*CYP2D6* phenotype			
IM	0.105 (−0.064 to 0.275)	.221	3.17
NM + UM	0.210 (0.030 to 0.391)	.023	

Note

Total *R* ^2^: **51.01;** MAE = 0.16 ng ml‐1; MAPE = 19.94%.

**Table 4:** β Coefficients and significance levels of variables significantly associated with log‐transformed ENDOss, after substituting Log(DM/DX) for *CYP2D6* phenotype in multivariable regression analysis (Equation 2)

Variable	β Coefficient (95% CI)	*P*‐value	Partial *R* ^2^
Intercept	0.225 (0.023 to 0.427)	.030	—
Log(DM/DX)	−0.223 (−0.262 to −0.184)	<.0001	39.25
Age (years)	0.0065 (0.003 to 0.009)	<.0001	5.13
Weak inhibitor use	−0.235 (−0.377 to −0.092)	.001	3.46

Note

Total *R* ^2^: **47.84;** MAE = 0.16 ng ml‐1; MAPE = 21.19%.

**Table 5:** β Coefficients and significance levels of variables significantly associated with log‐transformed ENDOss, after removing log(DM/DX) from multivariable regression analysis (Equation 3)

Variable	β Coefficient (95% CI)	*P*‐value	Partial *R* ^2^
Intercept	0.218 (−0.009 to 0.445)	.060	—
*CYP2D6* phenotype			
IM	0.444 (0.310 to 0.577)	<.0001	34.73
NM + UM	0.608 (0.480 to 0.735)	<.0001	
Weak inhibitor use	−0.265 (−0.418 to −0.112)	.001	3.47
Age (years)	0.0041 (0.001 to 0.007)	.010	1.86

Note

Total *R* ^2^: **40.96;** MAE = 0.17 ng ml‐1; MAPE = 21.79%.

**Table 6:** Mean absolute error (MAE) and mean absolute percentage error (MAPE) of ENDOss predictions obtained with the three models developed

	Equations (n°)		
	1	2	3
MAE (ng ml‐1)			
mean	3.76	3.89	4.07
SD	4.09	4.09	4.49
range	0.002‐24.19	0.018‐24.49	0.016‐27.69
MAPE (%)			
mean	39.74	41.84	43.53
SD	42.52	42.95	46.24
range	0.02‐252.59	0.18 −227.81	0.59‐239.69

## 4 DISCUSSION

This study showed that the co‐variables log(DM/DX), age and weak inhibitor use, and *CYP2D6* phenotype correlated significantly with log‐ENDOss on multiple regression analysis, explaining 51.0% of log‐ENDOss variability. The best predictor was log(DM/DX) (partial *R* ^2^ = 0.39), with the contributions of age (partial *R* ^2^ = 0.051), weak inhibitor use (partial *R* ^2^ = 0.035), and *CYP2D6* phenotype (partial *R* ^2^ = 0.032) decreasingly important (Table 3). When *CYP2D6* phenotype was removed from the regression, the overall *R* ^2^ was marginally lower (0.48), whereas removing log(DM/DX) resulted in a greater decrease in *R* ^2^ (0.41).

These results were not unexpected because the expression of *CYP2D6* activity is controlled by several nongenetic factors,^12^ which matter especially in patients with intermediate‐to‐fast genotypes. In fact, 34% of IMs, NMs and UMs did not match the phenotype derived from log(DM/DX), whereas only 1 of 14 PMs was classified as IM by log(DM/DX) (Figure 3).

Other potential limitations of the activity score system are that not all *CYP2D6* variant alleles are routinely genotyped and that the scoring criteria may change as new information becomes available. Indeed, the score has been challenged by Schroth et al,^23^ who showed that downgrading CYP2D6*10 activity score from 0.5 to 0.25 improved ENDOss prediction, so new guidelines have recently been updated.^21^ In short, phenoconversion and activity score misclassification can both weaken the predictive power of *CYP2D6* genotype. On the other hand, genotype is stable for life, whereas DM levels may change due to intervening, non‐genetic factors (Table 7).

**Table 7:** Pros and cons of the two methods used for phenotyping *CYP2D6* activity

	PROs	CONs
Log(DM/DX) metabolic ratio	The influence of non‐genetic factors (drug‐drug interactions, co‐morbidities, pregnancy, etc) is included	Results may change over time Renal function and urine pH may affect the log(DM/DX) metabolic ratio Time‐consuming (urine collection, drug/metabolite assay)
*CYP2D6* phenotype (CPIC)	Single blood sample required Genotype does not change over time	Not all *CYP2D6* variants are routinely assessed The activity score attributed to each variant allele may be challenged Phenoconversion can bias the results

Several clinical studies investigated the correlation between *CYP2D6* genotype‐derived activity and ENDOss, using various methods and phenotyping criteria, and with mixed results. In general, individuals labelled as PMs have significantly lower ENDOss than EMs.^24^ Only four studies phenotyped *CYP2D6* activity with the dextromethorphan test, using different experimental approaches. De Graan et al ^25^ calculated the area under the concentration‐time curve of DM during a 6‐hour interval in 40 women, finding it correlated inversely with trough ENDOss (*r* = −0.72). Opdam et al,^26^ and Safgren et al ^27^ used the ^13^C‐DM breath test, measuring the expired ^13^CO_2_ as an index of DM demethylation: they reported significant correlations between the changes in ^13^CO_2_ and ENDOss, with r values of 0.56 (n = 77) and 0.69 (n = 65), respectively. Antunes et al ^28^ simultaneously phenotyped *CYP2D6* and *CYP3A4* activities in 116 patients by calculating the [DM]/[DX] and [omeprazole]/[omeprazole sulfone] metabolic ratios in a single plasma sample obtained 3 hours after oral administration of DM and omeprazole. They found that the [DM]/[DX] ratio was associated with ENDOss (r = −0.52), but the [omeprazole]/[omeprazole sulfone] ratio was not.

Incidentally, our phenotyping method based on the urinary log(DM/DX) ratio was validated by comparison with the reference debrisoquine test ^10^ and found to correlate with the partial metabolic clearance of DM to DX.^11^ A good correlation was also demonstrated in our population between urinary log(DM/DX) ratio and plasma log(ND‐TAM/ENDO) ratio, which is another measure of *CYP2D6* activity (Figure 5). Notably, Saladores et al ^5^ reported that the ND‐TAM/ENDO metabolic ratio correlated significantly with the RFS hazard ratio (HR) on multivariable Cox's regression.

Efforts to predict ENDOss may be justified to the extent that they can forecast treatment outcomes. Conflicting data are available for now. Madlensky et al,^4^ and Saladores et al ^5^ documented better outcomes when ENDOss plasma concentrations exceeded 14‐16 nM (5.2‐5.97 ng ml‐1). It should be noted that both studies included women with early‐stage breast cancer and Saladores's study only considered premenopausal patients. Another study on a small sample (n = 86) with a long median follow‐up (13.8 years) found long‐term overall survival worse for patients with ENDOss concentrations <9 nM or 4OH‐TAM <3.26 nM.^29^ The CYPTAM study showed that neither *CYP2D6* genotypes nor ENDOss levels were associated with RFS in 667 women taking TAM (20 mg q.d.) ^30^ for a median of 2.5 years (median follow‐up 6.4 years), then shifted to an aromatase inhibitor in 66% of cases. Given the long time to recurrence of breast cancer, the short duration of therapy and follow‐up may explain the negative results of this study. In addition, the HR used to estimate the sample size was probably too high as well (HR = 2), compared with the HR of 1.4 found significant in Madlensky's study. Two other studies found no association between ENDOss and clinical endpoints.^31^, ^32^ All patients had advanced breast cancer, however, and most of them were post‐menopausal. The conflicting results may therefore be due to differences in patient selection (cancer stage, menopausal status) and study design (duration of therapy and follow‐up, sample size calculation). In agreement with this hypothesis, Margolin et al ^33^ reported that *CYP2D6* genotypes with low activity scores (presumably associated with low ENDO levels) had negative outcomes mainly in pre‐menopausal women, and suggested that higher estrogen levels require a more efficient TAM bio‐activation. In the same vein, advanced cancer stages may be less responsive to anti‐estrogen therapy, thus yielding a flat correlation between ENDOss and clinical outcomes.

While waiting for more conclusive results, it has been suggested that TAM dose be adjusted according to ENDOss rather than *CYP2D6* geno‐phenotype.^29^, ^34^, ^35^ In line with this view, Fox et al ^36^ increased the TAM doses in 68 of 122 patients based on their individual ENDOss levels. Following this dose escalation, the percentage of patients with ENDOss >15 nM rose from 76% to 96% and the percentage of those with ENDOss >30 nM from 34% to 76%. Two other studies showed that TAM dose escalation from 20 mg to 40 mg q.d. did not increase the frequency or severity of side effects.^37^, ^38^

This approach seems appealing, but means that TAM dosage can only be adjusted after 2‐3 months of therapy, when a steady state has been reached.

An alternative strategy—suggested by Hertz and Rae,^39^ and supported by our results—could reduce the time it takes to optimize the TAM dosage. Before starting treatment, we can compute patients’ log(DM/DX) or CYP2D6 phenotype (depending on the method available at the point of care), and predict their log‐ENDOss using Equation 2 or Equation 3, then obtain their ENDOss by calculating the anti‐logarithm.

Whatever the method used, patients whose predicted ENDOss concentration is lower than the threshold of 5.97 ng mL‐1 should start therapy with doses >20 mg. Assuming a linear dose‐concentration relationship, the dose should be increased by the threshold‐to‐predicted‐concentration ratio and rounded up to 30 or 40 mg. Should higher (off‐label) doses be required, aromatase inhibitors may be an alternative option, since little is known about the long‐term safety of higher doses of TAM.^36^

Adequately‐powered prospective trials are obviously needed to test this strategy.

Our study has some limitations. First, we assumed that all patients adhered to their TAM treatment. Though we could not prove as much, it is reasonable to assume a good compliance at the start of the therapy. The long half‐lives of TAM and its metabolites should also guarantee stable ENDO concentrations even if a TAM dose is missed occasionally. Second, we did not genotype all the known CYP2D6 variants, but only those most common in Caucasians, so our results cannot be extended to other ethnicities. Third, ENDOss are reportedly 20% lower in winter than the mean year‐round levels.^40^ Our study covered a period of 8 months, so ENDOss may have been influenced by seasonal changes. That said, a post‐hoc analysis of our data (not shown) found no differences in ENDOss measured in January‐March versus July‐September. Fourth, the results of urinary DM testing may be affected by changes in urinary pH and renal function, thus leading to misphenotyping *CYP2D6*. Although we cannot exclude this possibility, such a bias can be minimized by collecting urine over a long period (10 hours), as we did. The log(DM/DX) ratio also correlated strongly with the log(ND‐TAM/ENDO), which more closely reflects ENDO production by *CYP2D6*.

## 5 CONCLUSIONS

Our study found that phenotyping *CYP2D6* activity by means of a urinary DM test is the single best predictor of ENDOss. A model including log(DM/DX), patient's age, and use of *CYP2D6* inhibitors has an acceptable predictive performance, and could be used as an alternative to genotyping tests. Despite some uncertainty regarding the optimal ENDOss, a therapeutic approach that aims at personalizing TAM dosage early on is worth testing in a prospective trial.

## CONFLICT OF INTERESTS

None of the authors have any competing interests to disclose.

## AUTHORS’ CONTRIBUTIONS

MG, FP, NM, and RP contributed to study conception and design. LB, GDR, CF, YM, CB, DDC, APF, ST, EC, AB, CM, and RS contributed to data acquisition. MG, BC, NM, and RP contributed to data analysis and interpretation. MG, BC, and RP contributed to manuscript drafting. MG, FP, BC, CO, NM, and RP contributed to manuscript revision.

## Supporting information

Supplementary Material

## Data Availability

The data analyzed in this study are available from the corresponding author on reasonable request.

## Appendix 1

The Italian TAM Study Group

The following people contributed to this study:

Silvia Angelini^5^, Susanne Baier^10^, Carmen Barile^4^, Franco Bassan^20^, Emanuela Beda^13^, Laura Bertolaso^4^, Andrea Bonetti^16^, Lucia Borgato^1^, Antonella Brunello^1^, Maria Paola Cecchini^16^, Barbara Corso^21^, Giorgio Crepaldi^4^, Elisabetta Cretella^10^, Donatella Da Corte Z.^9^, Cristina Dealis^10^, Giovanni DeRosa^22^, Emilia Durante^16^, Cristina Falci^2^, Adolfo Favaretto^5^, Elena Fiorio^7^, Anna Paola Fraccon^6^, Lara Furini^12^, Alice Giacobino^3^, Tommaso Giarratano^2^, Carlo Alberto Giorgi^2^, Filippo Greco^16^, Milena Gusella^4^, Alessandro Inno^4^, Micaela Lenotti^7^, Maria Rita Lusso^10^, Marta Mandarà^19^, Daniela Menon^4^, Federica Merlin^19^, Marta Mion^13^, Nadia Minicuci^21^, Yasmina Modena^4^, Caterina Modonesi^14^, Linda Nicolardi^20^, Cristina Oliani^4^, Roberto Padrini^22^, Dario Palleschi^5^, Felice Pasini^6^, Maria Cristina Pegoraro^17^, Elena Pellegrinelli^4^, Elisa Perfetti^3^, Elisa Pezzolo^4^, Paolo Piacentini^16^, Valentina Polo^5^, Concetta Raiti^18^, Elvira Rampello^19^, Ilaria Rocco^21^, Romana Segati^15^, Elena Seles^3^, Mariella Sorarù^13^, Laura Stievano^4^, Ottaviano Tomassi^18^, Silvia Toso^8^, Francesca Vastola^14^, Emanuela Vattemi^10^, Alberto Zaniboni^11^, Marta Zaninelli^12^

^1^Oncology Unit 1, Istituto Oncologico Veneto (IOV), IRCCS Padova, Italy; ^2^Oncology Unit 2, Istituto Oncologico Veneto (IOV), IRCCS Padova, Italy; ^3^Oncology Unit, Ospedale degli Infermi, Biella, Italy; ^4^Oncology Unit, Ospedale di Rovigo, AULSS5 Polesana, Italy; ^5^Oncology Unit, Ospedale di Treviso, AULSS2 Marca Trevigiana, Italy; ^6^Oncology Unit, Casa di Cura Pederzoli, Peschiera del Garda, Italy; ^7^Oncology Unit, AOUI Verona, Italy; ^8^Oncology Unit, Ospedale di Adria, AULSS5 Polesana, Italy; ^9^Oncology Unit, Ospedale di Belluno, AULSS1 Dolomiti, Italy; ^10^Oncology Unit, Ospedale di Bolzano, Az. Sanitaria dell'Alto Adige, Italy; ^11^Oncology Unit, Fondazione Poliambulanza, Brescia, Italy; ^12^Oncology Unit Ospedale di Villafranca, AULSS9 Scaligera, Italy; ^13^Oncology Unit, Ospedale di Camposampiero e Cittadella, AULSS6 Euganea, Italy; ^14^Oncology Unit, Ospedali Riuniti Padova Sud, AULSS6 Euganea, Italy; ^15^Oncology Unit, Ospedale di Feltre, AULSS1 Dolomiti, Italy; ^16^Oncology Unit, Ospedale di Legnago, AULSS 9 Scaligera, Italy; ^17^Oncology Unit, Ospedale di Montecchio Maggiore, AULSS8 Berica, Italy; ^18^Oncology Unit, Ospedale di S Dona', AULSS4 Veneto Orientale, Italy; ^19^Oncology Unit, Ospedale di San Bonifacio, AULSS9 Scaligera, Italy; ^20^Oncology Unit, Ospedale Altovicentino, AULSS 7 Pedemontana, Italy; ^21^National Research Council, Neuroscience Institute, Padova, Italy; ^22^Clinical Pharmacology Unit of the Department of Medicine (DIMED), University of Padova, Padova, Italy
