# Supplementary material for: Predicting steady‐state endoxifen plasma concentrations in breast cancer patients by CYP2D6 genotyping or phenotyping. Which approach is more reliable?
Source: Pharmacol Res Perspect. 2020 Aug 19;8(5):e00646. doi: 10.1002/prp2.646 (PMC7437348; doi:10.1002/prp2.646)
Supplement: Supplementary file 1 — Supplementary Material [file PRP2-8-e00646-s001.docx]

Appendix 1. Precision, accuracy and lower quantification limits of the analytical methods used.

| Analyte | Concentration (ng/mL) | Precision (%) | Accuracy (%) | LQL (ng/mL) |
| --- | --- | --- | --- | --- |
|  | | | | |
| ENDO | 1.25 | 7.6 | 110.2 | 1.25 |
|  | 20 | 2.8 | 100.7 |  |
| 4OH-TAM | 0.5 | 8.8 | 112.0 | 0.625 |
|  | 5 | 1.0 | 101.5 |  |
| ND-TAM | 100 | 1.9 | 95.2 | 25 |
|  | 400 | 1.3 | 100.4 |  |
| TAM | 100 | 2.1 | 97.4 | 25 |
|  | 400 | 1.6 | 98.6 |  |
|  | | | | |
| DM | 1000 | 9.9 | 105.6 | 16.3 |
|  | 10,000 | 6.2 | 98.7 |  |
| DX | 1000 | 4.0 | 113.1 | 28.7 |
|  | 10,000 | 2.7 | 105.6 |  |
